# In Vivo Monitoring of *Fabp7* Expression in Transgenic Zebrafish

**DOI:** 10.3390/cells13131138

**Published:** 2024-07-02

**Authors:** Sol Pose-Méndez, Michel Rehbock, Alexandra Wolf-Asseburg, Reinhard W. Köster

**Affiliations:** Cellular and Molecular Neurobiology, Zoological Institut, Technische Universität Braunschweig, 38106 Braunschweig, Germany; m.rehbock@tu-braunschweig.de (M.R.); alex.wolf@tu-braunschweig.de (A.W.-A.)

**Keywords:** zebrafish, radial glia, cell progenitors, oligodendrocytes, neurons, *Fabp7*, transgenic line, cerebellum, Bergmann glia

## Abstract

In zebrafish, like in mammals, radial glial cells (RGCs) can act as neural progenitors during development and regeneration in adults. However, the heterogeneity of glia subpopulations entails the need for different specific markers of zebrafish glia. Currently, fluorescent protein expression mediated by a regulatory element from the *glial fibrillary acidic protein* (*gfap*) gene is used as a prominent glia reporter. We now expand this tool by demonstrating that a regulatory element from the mouse *Fatty acid binding protein 7* (*Fabp7*) gene drives reliable expression in *fabp7*-expressing zebrafish glial cells. By using three different *Fabp7* regulatory element-mediated fluorescent protein reporter strains, we reveal in double transgenic zebrafish that progenitor cells expressing fluorescent proteins driven by the *Fabp7* regulatory element give rise to radial glia, oligodendrocyte progenitors, and some neuronal precursors. Furthermore, Bergmann glia represent the almost only glial population of the zebrafish cerebellum (besides a few oligodendrocytes), and the radial glia also remain in the mature cerebellum. *Fabp7* regulatory element-mediated reporter protein expression in Bergmann glia progenitors suggests their origin from the ventral cerebellar proliferation zone, the ventricular zone, but not from the dorsally positioned upper rhombic lip. These new *Fabp7* reporters will be valuable for functional studies during development and regeneration.

## 1. Introduction

Radial glial cells (RGCs) play essential roles as a source for neural progenitors and glia subpopulations, as a scaffold for cell migration, and as a reservoir during regeneration. RGCs are derived from neuroepithelial cells (NECs) [1] and have been shown in mice and zebrafish to express several evolutionarily conserved RGC marker genes including Glial fibrillary acidic protein (Gfap), S100 calcium binding protein B (S100b), the estrogen-synthesizing enzyme Aromatase B, and Brain Lipid Binding Protein (Blbp)—also named Fatty acid binding protein 7 (Fabp7) [2,3,4,5]. Differing from mammalians, the production of new neurons from radial glial cells is maintained in multiple areas of the central nervous system (CNS) of zebrafish until and during adulthood, under both physiological and regenerative conditions [3].

Fatty acid binding protein 7 is involved in lipid metabolism and membrane synthesis, strongly binding to omega-3 polyunsaturated fatty acids, such as docosahexaenoic acid (DHA) [2], is expressed in RGCs at very early stages, and is co-expressed with well-established radial glia markers such as *gfap* and *aromatase* B [2,3,4,5].

The expression of *Fabp7* in teleosts (zebrafish) differs from other glial markers, such as the intermediate filament Glial fibrillary acidic protein (Gfap), since Gfap expression is activated only in RGCs, while Fabp7 is already expressed in the NECs, the RGC progenitors [6]. In some zebrafish studies, though, Gfap cells colocalizing with Nestin (another neuroepithelial cell marker) were detected, for example, in the embryonic spinal cord [7]. In fact, the transition of neuroepithelial cells to radial glial cells to mature glia is poorly defined based on marker gene expression in species different than mice. Therefore, further markers and in vivo tools to study radial glia are needed to better understand the developmental time course of glia in zebrafish.

Currently, the only regulatory elements for driving expression in radial glial cells in zebrafish are the enhancer of the *glial fibrillary acidic protein* (*gfap*), as the most commonly used glial specific enhancer, and *aromatase-B*, used only in few studies so far, which drive expression in *fabp7* expressing RGCs but not in NECs [8]. *gfap* mRNA and Gfap protein expression show similar patterns, but it has also been reported that Gfap expression could be regulated at the level of translation, and cells producing *gfap* mRNA may not translate it, which could lead to misinterpretations about the glial identity of a cell [9,10].

The RGC marker Fabp7 is involved in brain development and adult neurogenesis [11], as well as in the maintenance of neural stem cell fate, acting as a downstream target of Notch signaling and Pax6 [12,13]. The Fabp7 protein is encoded by the gene *brain-type fatty acid binding protein 7*—*Fabp7* [12]. Due to the genomic duplication that occurred in teleost fish [14], the *fabp7* gene in zebrafish was duplicated to *fabp7a* and *fabp7b*. According to the levels of mRNA expression, *fabp7a* was determined to be the main isoform present in the adult zebrafish brain [2,11]. The specificity of the antibody against Fabp7 and its distribution in the whole brain of adult zebrafish has already been described in detail [2]. Thus, this RGC marker has been commonly used in multiple zebrafish studies about glio- and neurogenesis [4,7]. As informative as such immunohistochemical studies are, they are limited to fixed tissue and provide static information but do not allow for in vivo observations of *fabp7*-expressing cells and their descendants for assessing their behavior in longitudinal studies.

We have tested a *Fabp7* regulatory element from a mouse, which mediates expression in radial glia, immature astrocytes, and Bergmann glia [13,15]. This regulatory element was combined with an adenoviral basal promoter (E1b) [16] in zebrafish. Three different transgenic reporter lines for detecting cells expressing fluorescent proteins under the control of the mouse *Fabp7* regulatory element in zebrafish were created, and their prominent fluorescent reporter protein expression confined to the central nervous system was compared to endogenous Fabp7 and early gliogenic and neurogenic markers. These lines will be powerful genetic tools for dissection brain development, glia differentiation, neurogenesis, and regeneration.

## 2. Materials and Methods

Fish husbandry: All animals were raised and kept in the zebrafish facility in accordance with established guidelines adhering to the regulations of the local government [17,18]. AB wild-type or *brass* pigmentation mutant zebrafish were used for mating. No selection criteria were used to allocate zebrafish of both sexes to any experimental group. Embryos and larvae were maintained in zebrafish 30% Danieau rearing medium [100% Danieau: 58 mM NaCl, 0.7 mM KCl, 0.4 mM MgSO_4_, 0.6 mM Ca(NO_3_)_2_, and 5 mM HEPES (N-2-hydroxyethylpiperazine-N-2-ethane sulfonic acid), pH7.2]. All imaging and analysis of larvae were performed at 2 and 5 days post-fertilization (dpf). Adults were maintained in fish tanks according to standard protocols at 28 °C, under light–dark conditions (simulating day-night cycle), and constant running water exchange. Stable transgenic reporter lines used in this work include Tg(*Mmu.Fabp7-E1B*:MScarlet-MYC)^bz23^, Tg(*Mmu.Fabp7*:*E1B*:mClover)^bz25^ and Tg(*Mmu.Fabp7-E1B*:FMA-mseCFP-2A-H2B-mseCFP)^bz24^, approved by the Niedersächsisches Landesamt für Verbraucherschutz und Lebensmittelsicherheit LAVES (AZ 33.19-42502-04-17/2693), Tg(*gfap*:EGFP)^mi2001^ [19], Tg(*olig2*:EGFP)^vu12^ [20], Tg(*nkx2.2*:GFP) [21], Tg(*Xla.Tubb:DsRed, previously NBT-dsRED*) [22], Tg(zic4:Gal4TA4, UAS:mCherry)^hzm5^, Tg(4xUAS:GFP)^hzm3^ both by [23], Tg(*ptf1a*:EGFP) [24], and Tg(*atoh1a*:GAL4TA4)^hzm2^ [25]. For simplicity reasons double transgenic Tg(*atoh1a*: GAL4TA4)^hzm2^ × Tg(4xUAS:GFP)^hzm3^ larvae were named Tg(*atoh1a*:GFP). All efforts were made to use only the minimum number of experimental animals necessary to obtain reliable scientific data.

Generation of transgenic lines: Previously a regulatory element from the mouse *Fabp7* gene driving expression in radial glial cells has been identified and analyzed in detail [15,26]. This sequence of the mouse *Fabp7* regulatory element followed by the E1b basal promoter is shown in Supplementary Figure S1. mClover3 [27] and mScarlet [28] were chosen as fluorescent reporters as they are among the brightest fluorescent proteins available in the green and red emission range, mseCFP is a derivative of ECFP [29], but it can be excited as well in the UV range (405 nm) offering co-expression analysis with a greater range of fluorescent reporter proteins. Zebrafish embryos were injected at the one-cell stage with plasmids containing a Tol2-transgene cassette and Tol2-encoding mRNA (25 ng/l each, volume: 1.5 nl). After raising fluorescent embryos to adulthood, germline transmission of the transgene was tested by individual crosses against wild-type fish. Transgenic founders were raised and used to establish stable transgenic strains [30]. Reporter fish with fluorescent protein expression mediated by the *Fabp7* regulatory element are currently maintained in the F2 and F3 generations, respectively.

Immunohistochemistry: Larvae were processed for immunostaining as whole mount isolated brains. Brain isolation was performed after prefixing in 4% paraformaldehyde (PFA) for 20–30 min on ice. After washing with PBS, antigen retrieval with 10 mM citrate buffer was achieved by heating samples in a water bath until boiling. After cooling to room temperature, the brains were rinsed 5 times in PBS-T (PBS with 1% Triton X-100) for 5 min each, incubated in 100% precooled acetone for 15 min at −20 °C to improve tissue permeabilization, followed by additional 5 PBS-T washing steps. Nonspecific protein binding sites were blocked in 5% normal goat serum in PBS-DT-I (PBS, 1% bovine serum albumin (BSA), 1% DMSO, 1% Triton X-100) for 1 h at room temperature followed by primary antibody incubation in PBS-DT-II (PBS, 1% BSA, 1% DMSO, 0,3% Triton X-100) overnight at 4 °C and additional 3 h at room temperature. The primary antibodies are as follows: rabbit anti-FABP7 (IF 1:500, commercially named as anti-BLBP, Millipore ABN14, Burlington, MA, USA), rat anti-RFP—for detecting mScarlet fluorescent protein—(IF 1:1000, Chromotek 5F8-100, Planegg, Germany), and chicken anti-GFP (IF 1:1000, Aves Labs GFP-1010, Davis, CA, USA). Subsequently, brains were washed 5 times in PBS-T for 5 min each at room temperature with constant agitation and incubated with the secondary antibody diluted in PBS-DT-II overnight at 4 °C. Secondary antibodies: Alexa goat anti-rabbit 488 (IF 1:1000, Thermo Fisher A21206, Waltham, MA, USA), Alexa goat anti-rat 546 (IF 1:1000, Thermo Fisher A11081, Waltham, MA, USA), donkey anti-chicken (IF 1:1000, Jackson ImmunoResearch, 703-545-155, West Grove, PA, USA). Finally, brains were thoroughly rinsed in PBS-T and PBS before imaging.

Imaging and data analysis: For in vivo imaging, larvae were anesthetized with 0.015–0.02% Tricaine (Sigma Aldrich, St. Louis, MO, USA) dissolved in 1% low melting agarose/Danieau solution. For fixed and immunostained samples, the brains were embedded in 1% low melting agarose/PBS. Images of the larval zebrafish (at 2 and 5 dpf) were acquired using an SP8 laser scanning confocal microscope (Leica Microsystems, Wetzlar, Germany) using a 40× objective. Images were processed with the Leica LasX (LAS X 3.5.7.23225) and FIJI imaging (ImageJ-win64) and analysis software. For quantitative analysis, semi-automated counting of cells was performed with the cell counter plugin of the FIJI software, in which every cell was initially identified manually, followed by their automated detection within a z-stack of images, to avoid that cells were counted twice.

The design of the figures was carried out with CorelDraw software (Corel Corporation Ottawa, Ontario, Canada). Statistical analysis and graphics design were undertaken using Prism software (Graph Pad Software, Version 6, and 9.0.0 (121), San Diego, CA, USA).

## 3. Results

### 3.1. Generation and Characterization of Transgenic Reporter Lines Expressing Fluorescent Proteins under Control of a Mouse Fatty Acid Binding Protein 7 (Fabp7) Derived Regulatory Element

**Figure 1:**
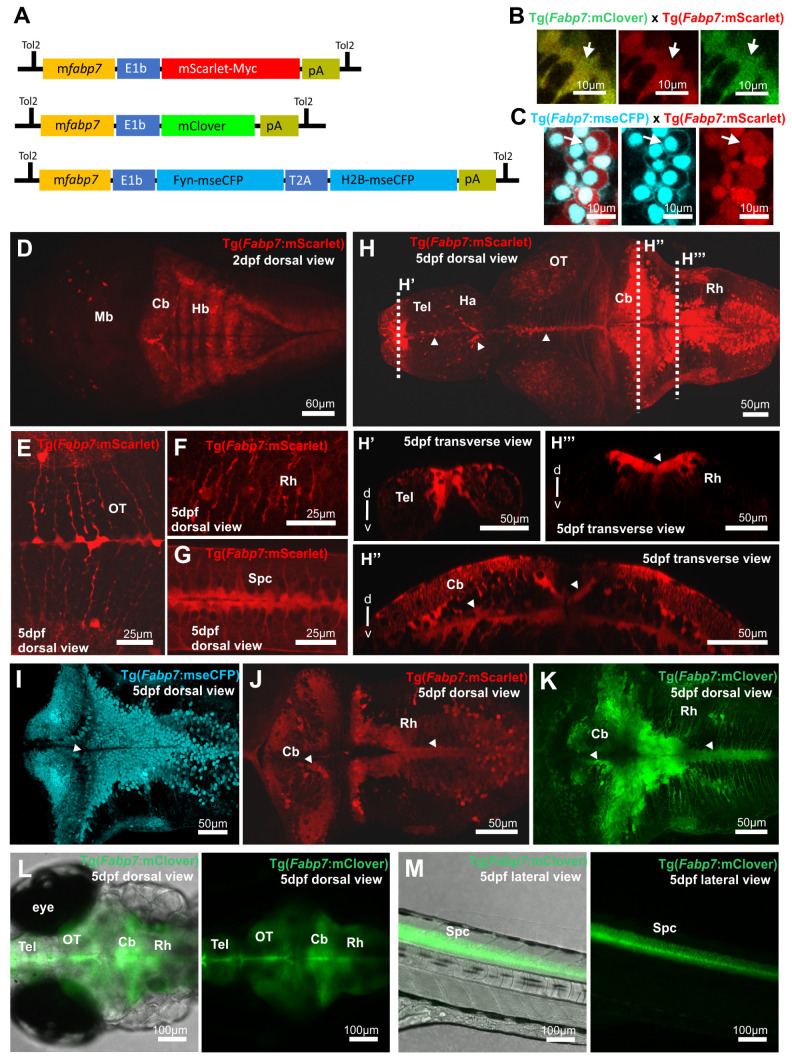
Transgenic zebrafish reporter lines at two and five days post-fertilization (dpf) expressing fluorescent reporter proteins under control of a mouse *Fatty acid binding protein 7* (*Fabp7*) regulatory element. (**A**) Schematic representation of the constructs from the transgenic reporter lines [Tg(*Fabp7*:mScarlet), Tg(*Fabp7*:mClover), and Tg(*Fabp7*:mseCFP)]. (**B**,**C**) Detail pictures of the double transgenic larvae Tg(*Fabp7*:mScarlet) × Tg(*Fabp7*:mClover) and Tg(*Fabp7*:mScarlet) × Tg(*Fabp7*:mseCFP), showing mScarlet and mClover expression in the cytoplasm (**B**,**C**), and mseCFP expression in the nucleus and cell membrane (**C**). (**D**–**M**) Representative overview (**D**,**H**,**I**–**M**) and detail (**E**–**G**,**H’**–**H’’’**) images showing the *Fabp7*-regulatory element mediated fluorescent protein expression pattern throughout the whole central nervous system from dorsal (**E**–**G**), lateral (**M**), and transverse view (**H’**–**H’’’**). Arrows point to double positive cells. Rostral is to the left. Abbreviations: *Fabp7 Fatty acid binding protein 7* regulatory element, Tel telencephalon, Ha habenula, Cb cerebellum, HB hindbrain, MB midbrain, OT optic tectum, Rh rhombencephalon, Spc spinal cord.

For the generation of transgenic lines, a previously identified 750 bp regulatory element derived from the mouse *Fabp7* genomic region [26] was cloned in front of the adenoviral basal promoter E1b [16]. This was followed by the open reading frame either of the green fluorescent protein mClover [27], the red fluorescent protein mScarlet [28] fused to a myc tag sequence at its C-terminus, or a combination of membrane targeted monomeric super-enhanced CFP (mseCFP) [29] Fyn-mseCFP followed by a self-cleaving T2A-sequence [31] and a nuclear-localized Histone2B-fusion of mseCFP (H2BmseCFP). Transgenic fish strains were obtained by established Tol2-transposon protocols [30] (Figure 1A).

While the transgenic reporter lines Tg(*Mmu.Fabp7-E1B*:MScarlet-MYC)^bz23^ [abbreviated name: Tg(*Fabp7*:mScarlet)] and Tg(*Mmu.Fabp7-E1B*:mClover)^bz25^ [abbreviated name: Tg(*Fabp7*:mClover)] express the mScarlet and mClover fluorescent protein in the cytoplasm (Figure 1B), mseCFP expression in the transgenic line Tg(*Mmu.Fabp7-E1B*:FMA-mseCFP-2A-H2B-mseCFP)^bz24^ [abbreviated name: Tg(*Fabp7*:mseCFP)] is localized to the cell membrane and the nucleus respectively (Figure 1C). In all three lines, expression becomes visible already in the brain at early embryonic stages around 14 hpf, which is similar to *gfap* enhancer-mediated expression [19], and fluorescent cells at 30 hpf are distributed only in the central nervous system [32], which is extensively labeled through the whole brain and spinal cord of young larvae, in the three transgenic reporter lines (Figure 1D–K, for a direct comparison of the expression patterns in the CNS among the three established lines please see Supplementary Figure S2) reminiscent to antisense mRNA in situ hybridization against *fabp7a* and *fabp7b* in zebrafish [11,33,34]. It is noteworthy that cells with *Fabp7* enhancer-mediated fluorescent protein expression are enriched in the most medial areas (in the midsagittal plane along the ventricular zone), such as those located in the telencephalon, habenula, optic tectum, cerebellum, caudal rhombencephalon, and spinal cord (arrowheads in Figure 1H–K), and show a more dispersed cell density in parenchymal areas of the aforementioned brain structures (Figure 1H–K). The strongest expression combined with a higher cell density of *Fabp7* enhancer-mediated fluorescent protein-expressing cells appears located in the most rostral area of the telencephalon (Figure 1H, H’), in the caudal area of the cerebellum, and in dorsal-paramedian areas of the caudal rhombencephalon (Figure 1H–K). In the cerebellum, *Fabp7*-regulatory element-mediated expression can be found in radially oriented cells with a unique branching of their membrane process visible in the molecular layer—the most dorsal cell layer of the cerebellar cortex (Figure 1H,H’’,I–K). Long *Fabp7* enhancer-regulated fluorescent protein-expressing processes without branches are also observed in the lateral areas of the optic tectum and caudal rhombencephalon (Figure 1E–F,I–K).

### 3.2. Colocalization among Different Fluorescent Fabp7 Enhancer-Controlled Reporter Lines

The consistency of the expression pattern among the three different *Fabp7* enhancer-regulated reporter transgenic lines was tested by analyzing the level of colabeling of mScarlet, mClover, and mseCFP fluorescent proteins in respective double transgenic offspring.

**Figure 2:**
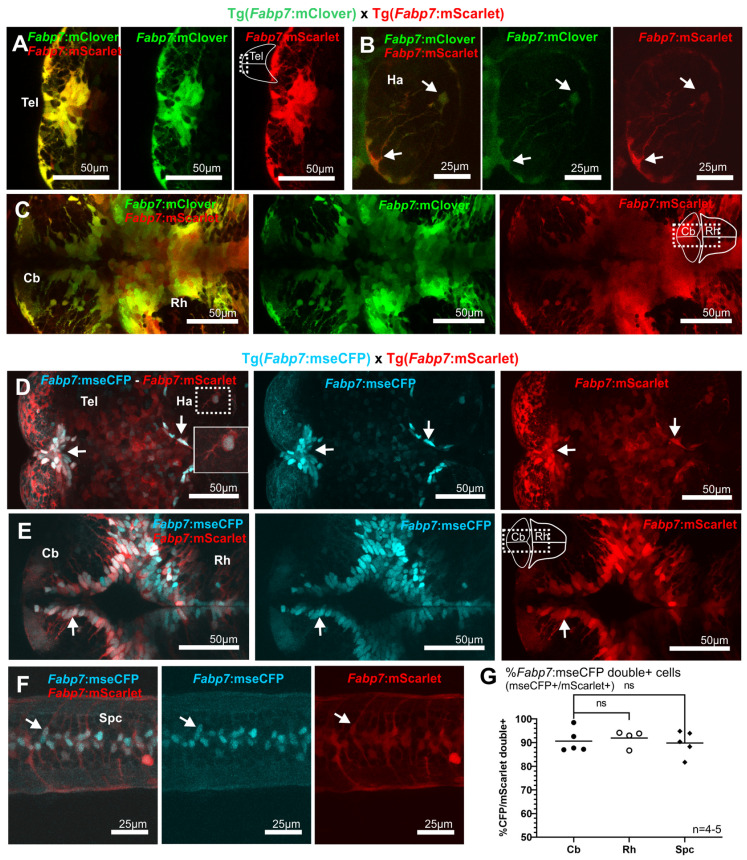
Double transgenic *Fabp7 enhancer-mediated* reporters in zebrafish larvae at 5 dpf. (**A**–**C**) Representative images of Tg(*Fabp7*:mScarlet) × Tg(*Fabp7*:mClover) double transgenic larvae showing the colocalization of both fluorescent proteins in the majority of cells. (**D**–**G**) Representative images (**D**–**F**) and quantitative analysis of double positive cells (**G**) in the Tg(*Fabp7*:mScarlet) × Tg(*Fabp7*:mseCFP) double transgenic larvae showing the colocalization of both fluorescent proteins in the majority of cells (average number and percentage of cells, corresponding to the quantification indicated in the graphs, are detailed in Supplementary Table S1). Arrows point to double positive cells. Dotted box indicates the area corresponding to detail picture (solid box) included in the figure. Rostral is to the left. Statistical method: Kruskal–Wallis multiple comparison test for nonparametric data was applied. Abbreviations: Cb cerebellum, ns no significant differences, Rh rhombencephalon, Spc spinal cord, Tel telencephalon.

Larvae derived from Tg(*Fabp7*:mScarlet) × Tg(*Fabp7*:mClover) and Tg(*Fabp7*:mScarlet) × Tg(*Fabp7*: mseCFP) crosses of heterozygous carriers showed largely overlapping expression patterns throughout various regions of the CNS (Figure 1H-M and Figure 2A–F), as illustrated in the telencephalon (rostral part in Figure 2A, D) habenula (mostly located in the marginal zone, and a few in the parenchymal area, Figure 2B,D), cerebellum (mostly in the area of the ventricular zone, Figure 2C,E), and spinal cord (Figure 2F), with the majority of cells double positive for the respective fluorescent proteins in 5 dpf larvae (arrows in Figure 2).

Three CNS areas were selected to support this observation by quantification. This analysis showed that in Tg(*Fabp7*:mScarlet) × Tg(*Fabp7*:mseCFP) double transgenic larvae, about 90% of cells expressing mseCFP also expressed mScarlet fluorescent protein without a significant difference between the cerebellum (90.6%), caudal rhombencephalon (91.9%), and spinal cord (89.8%), (Figure 2G). Of note, in order to test the consistency in the spatial expression of both lines, the percentage of the total mScarlet expressing cells double positive in the spinal cord was also quantified (93.4%), which did not appear significantly different from the total amount of mseCFP^+^ cells. That it could not be detected in all cell expressions of both fluorescent proteins likely depends on the different strength of fluorescence, with mseCFP being clearly weaker in its emission than mScarlet. In addition, the half-life of maturation and turnover of both reporter proteins may differ.

### 3.3. Specificity of Fabp7 Enhancer-Mediated Reporter Line Expression Pattern

**Figure 3:**
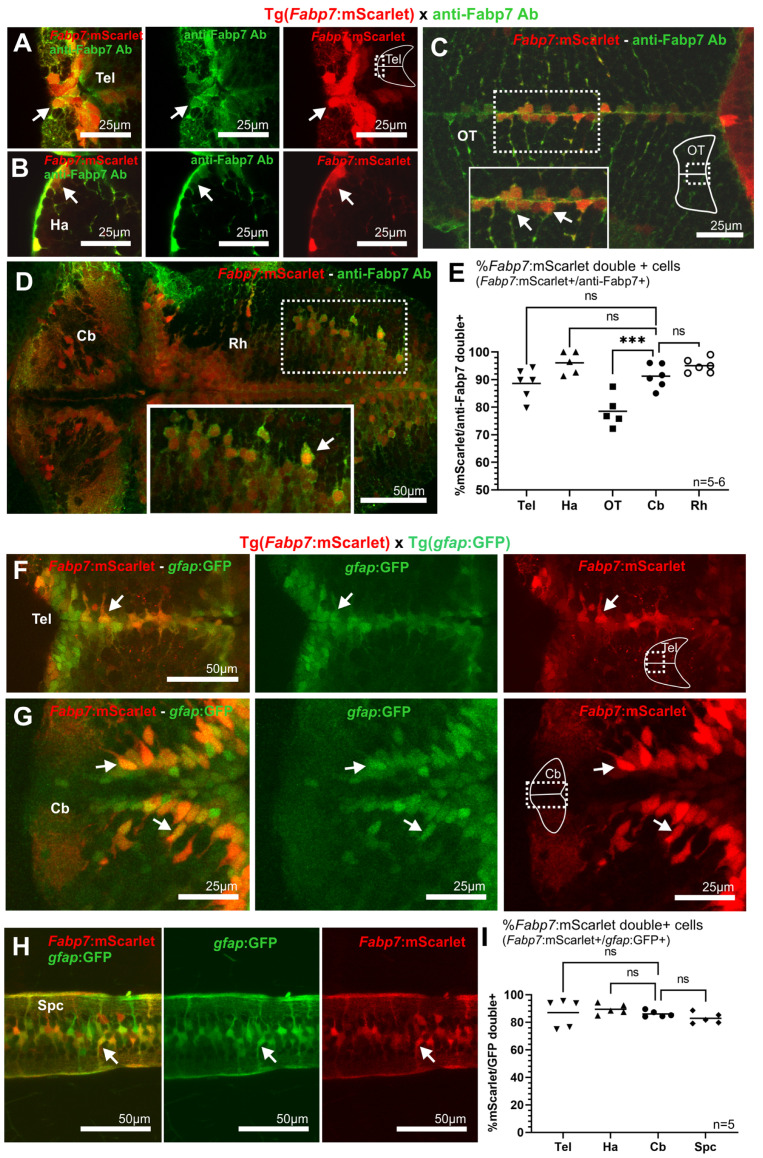
Comparison of the *Fabp7* enhancer-mediated reporter expression to anti-Fabp7 immunohistochemistry and radial glia expression in transgenic *gfap* enhancer-mediated reporter zebrafish larvae at 5 dpf. (**A**–**E**) Pictures (**A**–**D**) and quantitative analysis (**E**) of reporter protein expression in the transgenic line Tg(*Fabp7*:mScarlet) and the antibody anti-Fabp7. (**F**–**I**) Pictures (**F**–**H**) and quantitative analysis of double positive cells (**I**) in the double transgenic larvae Tg(*Fabp7*:mScarlet) × Tg(*gfap*:GFP) (average number and percentage of cells, corresponding to the quantification indicated in the graphs, are detailed in Supplementary Table S1). Arrows point to double positive cells. Dotted box indicates the area corresponding to detail pictures (solid box) included in the figures. Rostral is to the left. Statistical method: Ordinary one-way ANOVA, Sidák’s (**E**, for parametric or normal distribution data), and Kruskal–Wallis (**I**, for nonparametric data) multiple comparison tests were applied, level of significance: *p* < 0.001 (***). Abbreviations: Cb cerebellum, Ha habenula, ns no significant differences, OT optic tectum, Spc spinal cord, Tel telencephalon.

The specificity of the expression pattern of the transgenic line Tg(*Fabp7*:mScarlet) was tested by colocalization analysis of mScarlet with the endogenous Fabp7 protein using immunohistochemistry and commercially available antibodies (Figure 3A–E). The quantification of fluorescent cells (anti-mScarlet+/anti-Fabp7+) revealed that the vast majority of mScarlet-expressing cells were double positive co-expressing both proteins (telencephalon 88.6%, habenula 96%, optic tecum 78.5%, cerebellum 91.2%, and rhombencephalon 95%). Almost perfect colabeling was observed in the ventricular/subventricular zones, paramedian and lateral or parenchymal areas of all brain structures analyzed: telencephalon (rostro-medial zone, Figure 3A), habenula (marginal zone, Figure 3B), optic tectum (median area, Figure 3C), cerebellum and caudal rhombencephalon (paramedian area, Figure 3D). These findings support the specificity of the mouse *Fabp7* regulatory element mediating fluorescent reporter protein expression in cells expressing Fabp7 endogenously in zebrafish. A few exceptions, though, were noticed, for example, in the lateral areas of the optic tectum, where the percentage of colabeled cells was significantly lower compared to other brain regions for currently unclear reasons (Figure 3E).

The observation of about 10% of the cells being positive only for mScarlet could be due to the long half-life of fluorescent proteins, likely displaying a slower turnover compared to the Fabp7 protein. Yet, the possibility of ectopic expression in a few cells and/or insufficient fluorescence intensity detection of the anti-Fabp7 antibody into the tissue cannot be completely ruled out. Nevertheless, comparative analysis of the expression pattern in the transgenic *Fabp7* regulatory element-controlled reporter line with other specific cell type reporter lines might help to provide further confirmation of the specificity of the *Fabp7* enhancer-mediated expression pattern.

Altogether, the colocalization of the anti-Fabp7 antibody staining and *Fabp7*:mScarlet, as well as the overlapping expression pattern of the Tg(*Fabp7*:mScarlet) and Tg(*Fabp7*:mClover) and Tg(*Fabp7*:mseCFP) lines, suggest that the three reporter lines are indeed specific for cells expressing Fabp7 endogenously and can be used interchangeably.

### 3.4. Comparison of Fabp7 Enhancer-Regulated Reporter Lines to Other Cell Type-Specific Transgenic Reporter Lines

Comparison of transgenic fluorescent protein reporter strains helps to further characterize the spatio-temporal activity of the *Fabp7* enhancer-mediated fluorescent protein expression. We have therefore examined different areas of the brain and the spinal cord, with particular attention to the cerebellum, by comparing the expression of our *Fabp7* regulatory element-controlled reporter to fluorescent protein expression patterns in other cell type-specific reporter strains.

#### 3.4.1. Coexpression of *Fabp7-* and *gfap*-enhancer-Mediated Reporter Strains in Radial Glia

To further confirm the expression of the *Fabp7* regulatory element in radial glial cells, we compared *Fabp7* enhancer-mediated reporter protein expression to the expression of the green fluorescent protein in the Tg(*gfap*:GFP)^mi2001^ strain, in which a regulatory element of the *glial fibrillary acidic protein*-encoding gene (*gfap*) was shown to activate reporter protein expression in radial glia [19]. In the majority of *Fabp7*:mScarlet red fluorescent cells, colabeling by the green fluorescent protein GFP was observed at 5 dpf in double transgenic [Tg(*fabp7*:mScarlet) x Tg(*gfap*:GFP)] offspring. Comparison of the percentage of *Fabp7-gfap* mediated reporter protein colabeling of cells in the cerebellum (ventricular and subventricular zone, arrows in Figure 3G) and other brain regions and spinal cord (arrows in Figure 3F–H and Figure 7C), revealed similar degrees of cell colabeling in the telencephalon 87%, the habenula 89.5%, the cerebellum 86.1%, and the spinal cord 82.9% (Figure 3I and Figure 7A). The small number of some mScarlet single positive cells, like a few scattered cells in lateral areas of the telencephalon, might be an indication for the minor heterogeneity of *Fabp7* enhancer-mediated and *gfap* enhancer-controlled expression in the different radial glia and progenitor cell subpopulations.

#### 3.4.2. Oligodendrocytes: A Subset of *Fabp7*-Expressing Cells Become Oligodendrocytes

**Figure 4:**
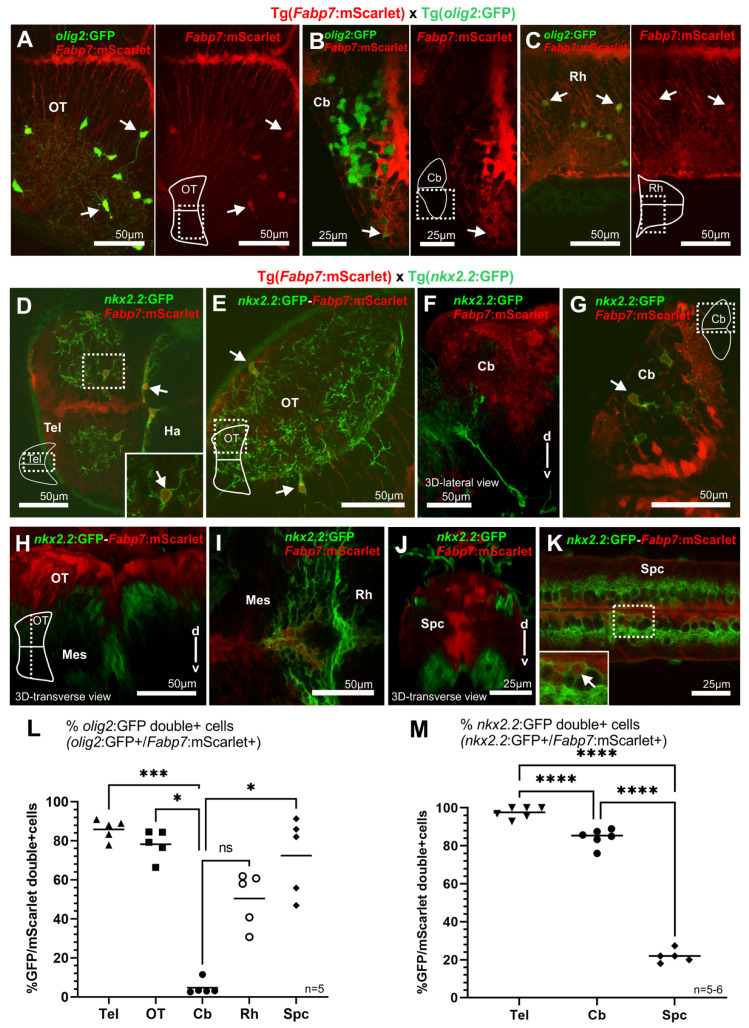
Comparison of the *Fabp7* enhancer-regulated reporter expression to transgenic *olig2* and *nkx2.2* oligodendrocyte zebrafish reporter strains at 5 dpf. (**A**–**C**,**L**) Pictures (**A**–**C**) and quantitative analysis (**L**) of the double transgenic larvae Tg(*Fabp7*:mScarlet) × Tg(*olig2*:GFP). (**D**–**K**,**M**) Pictures (**D**–**K**) and quantitative analysis of double positive cells (**M**) in the double transgenic larvae Tg(*Fabp7*:mScarlet) × Tg(*nkx2.2*:GFP) (**H**,**I**) (average number and percentage of cells, corresponding to the quantification indicated in the graphs, are detailed in Supplementary Table S1). Arrows point to double positive cells. Dotted box indicates the area corresponding to detail picture (solid box) included in the figure. Rostral is to the left. Statistical method: ANOVA test for multiple group comparison (ordinary one-way ANOVA followed by Šidák’s multiple comparisons test for parametric or normal distribution data (**M**) or Kruskal–Wallis (**L**) for nonparametric data), level of significance: *p* < 0.05 (*), *p* < 0.001 (***), *p* < 0.0001 (****). Abbreviations: Cb cerebellum, Ha habenula, Mes mesencephalon, ns no significant differences, OT optic tectum, Rh rhombencephalon, Spc spinal cord, Tel telencephalon.

In mice, *gfap*-expressing radial glia mostly differentiate into astrocytes [35]. While in zebrafish, glia with a typical astrocyte morphology, as observed in mammals, are not present, astrocyte-like cells have been identified in zebrafish [36]. Besides such astrocytic-like cells, glia progenitors also give rise to oligodendrocytes, of which the progenitors are known to express the transcription factors Olig2 and Nkx2.2a [21]. Double transgenic larvae from matings of the Tg(*Fabp7*:mScarlet) reporter line with carriers of the Tg(*olig2*:GFP) and Tg(*nkx2.2*:GFP) reporter strains revealed colabeling of cells with *Fabp*7 regulatory element-mediated fluorescent reporter protein expression with both oligodendrocyte reporters, mostly located in lateral/parenchymal areas (Figure 4A–K), in the telencephalon (arrow in paramedian zone, Figure 4D), optic tectum (arrows in paramedian and submarginal zones, Figure 4A,E), cerebellum (arrows in lateral areas, Figure 4B,G), and caudal hindbrain (arrows in lateral zone, Figure 4C). Quantification of *olig2* enhancer regulated GFP expressing cells co-expressing *Fabp7* regulatory element-mediated mScarlet expression revealed a high degree of GFP and mScarlet co-expression in the telencephalon 85.8%, the optic tectum 78.2%, and the spinal cord 72.4%, and about half of the population in the caudal rhombencephalon (Figure 3A–C,L and Figure 7A,C). Intriguingly, the cerebellum presented an exception with only 4.7% of *olig2* expressing cells co-expressing the *Fabp7* enhancer-regulated reporter (Figure 4B,L). This observation is due to the fact that not only oligodendrocytes are very low in numbers in the cerebellum, but also that *olig2* expression in the cerebellum is largely confined to a neuronal population of eurydendroid cells as the main cerebellar efferent neurons [37].

With respect to the oligodendrocyte reporter Tg(*nkx2.2*:GFP), double transgenic Tg(*Fabp7*:mScarlet) x Tg(*nkx2.2*:GFP) larvae and the majority of GFP-expressing cells were also positive for *Fabp7*:mScarlet expression with 97.6% of colabeled cells in the telencephalon and 84.4.% colabeled cells in the cerebellum (although representing only about 8% of the *Fabp7*:mScarlet cells colabeled with GFP, as the majority of *Fabp7*-enhancer expressing cells develop into radial glia and only a minor portion into oligodendrocytes, as it is the case in the telencephalon as well, Figure 7A) because *nkx2.2a* expression is confined to the oligodendrocyte lineage but is not expressed in *olig2*-expressing eurydendroid neurons. This suggests that oligodendrocyte progenitors are derived from *Fabp7* enhancer-controlled fluorescent protein-expressing glial cells (Figure 4D–K,M and Figure 7A,C). Of note, in all structures analyzed, the double positive cells were mainly found in lateral areas of the central nervous system, suggesting that these glial cells differentiating into oligodendrocytes are separated from the proliferative regions. In the spinal cord, only 21.8% of *nkx2.2*:GFP expressing cells co-expressed *Fabp7*:mScarlet with many solely *nkx2.2*:GFP positive cells being located in ventral positions. These cells may not give rise to oligodendrocytes but have been shown in mice and chicken embryos to be involved in ventral neural tube patterning being expressed in floorplate cells and later in somatic and visceral motoneurons [38,39,40].

#### 3.4.3. Neuronal Cells: Some *Fabp7* Regulatory Element-Mediated Fluorescent Protein Expressing Cells Are Neurogenic

**Figure 5:**
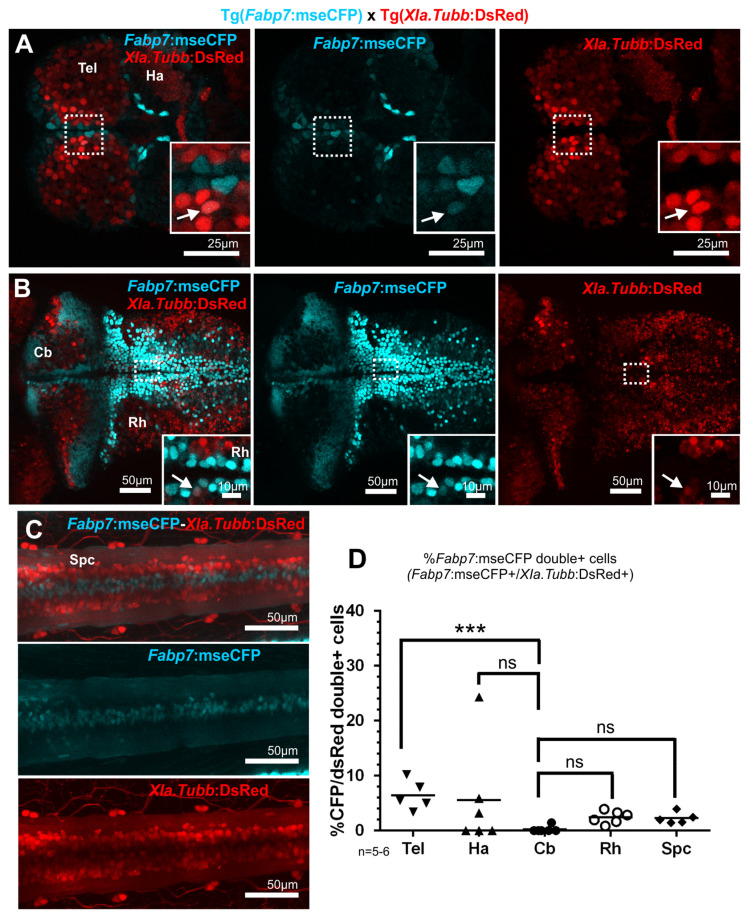
Comparison of the *Fabp7* reporter expression to transgenic *XIa.Tubb* pan-neuronal zebrafish reporter strain at 5 dpf. (**A**–**D**) Pictures (**A**–**C**) and quantitative analysis of double positive cells (**D**) in the double transgenic larvae Tg(*Fabp7*:mseCFP) × Tg(*XIa.Tubb*:DsRed) (average number and percentage of cells, corresponding to the quantification indicated in the graph, are detailed in Supplementary Table S1). Arrows point to double positive cells. Dotted box indicates the area corresponding to detail pictures (solid box) included in the figures. Rostral is to the left. Statistical method: Ordinary one-way ANOVA, Kruskal–Wallis multiple comparison test for nonparametric data was applied, level of significance: *p* < 0.001 (***). Abbreviations: Cb cerebellum, Ha habenula, ns no significant differences, Rh rhombencephalon, Spc spinal cord, Tel telencephalon.

The regulatory element derived from the *Xenopus laevis neural-specific beta-tubulin* gene mediates pan-neuronal expression of the fluorescent protein DsRed in the transgenic zebrafish strain Tg(*Xla.Tubb:DsRed)* [22]. The carriers of this strain were used to compare *Fabp7* regulatory element-derived expression to neuronal expression. The analysis of double transgenic larvae of the genotype Tg(*Fabp7*:mseCFP) × Tg(*Xla.Tubb:DsRed)* revealed mostly complementary and non-overlapping expression patterns since the *Fabp7* enhancer-mediated mseCFP-expressing cells were more concentrated in the ventricular areas, and *Xla.Tubb+* cells were mainly located in the paramedian and parenchymal areas of the telencephalon (Figure 5A), the cerebellum, the caudal rhombencephalon (Figure 5B), and the spinal cord (Figure 5C) (Figure 5A–C and Figure 7C). This supports the fact that the *Fabp7* regulatory element-induced expression is mostly confined to glial and not neuronal lineages.

However, at least a few *Fabp7 and XIa.Tubb* enhancer-mediated fluorescent proteindouble positive cells were detected (arrows in Figure 5), mostly located in the subventricular zone, as would be expected for recently differentiated neuronal cells. For instance, in the habenula (5.6% of *Fabp7*: mseCFP-cells were positive for DsRed), the caudal rhombencephalon (2.4%) and the spinal cord (2.3%) co-expression of both fluorescent proteins were displayed by a small subset of cells. This rate was lowest for the cerebellum (0.2%) and highest for the telencephalon (6.4%) (Figure 5D and Figure 7A). These findings of *Fabp7-Xla.Tubb* enhancer-mediated fluorescent protein double positive cells suggest that some *Fabp7* regulatory element-controlled fluorescent protein-expressing cells differentiate further into neurons, which can be observed due to the high stability and slow turnover of the mseCFP fluorescent protein compared to the endogenous Fabp7 protein detected by immunohistochemistry.

Indeed, different from immunohistochemistry with the anti-Fabp7 antibody, the mseCFP reporter allowed for temporarily tracing the offspring of at least some *Fabp7^+^* regulatory element-mediated fluorescent protein-expressing cells to develop into a neuronal fate, as at least a few differentiated neurons showed remaining mseCFP fluorescence. This finding could further explain the aforementioned small percentage of cells from the *Fabp7* regulatory element-regulated transgenic reporter line that was apparently negative for the anti-Fabp7 antibody (Figure 3E).

#### 3.4.4. *Fabp7* Regulatory Element-Mediated Fluorescent Protein Expression in Neural Progenitor Cells of the Hindbrain

**Figure 6:**
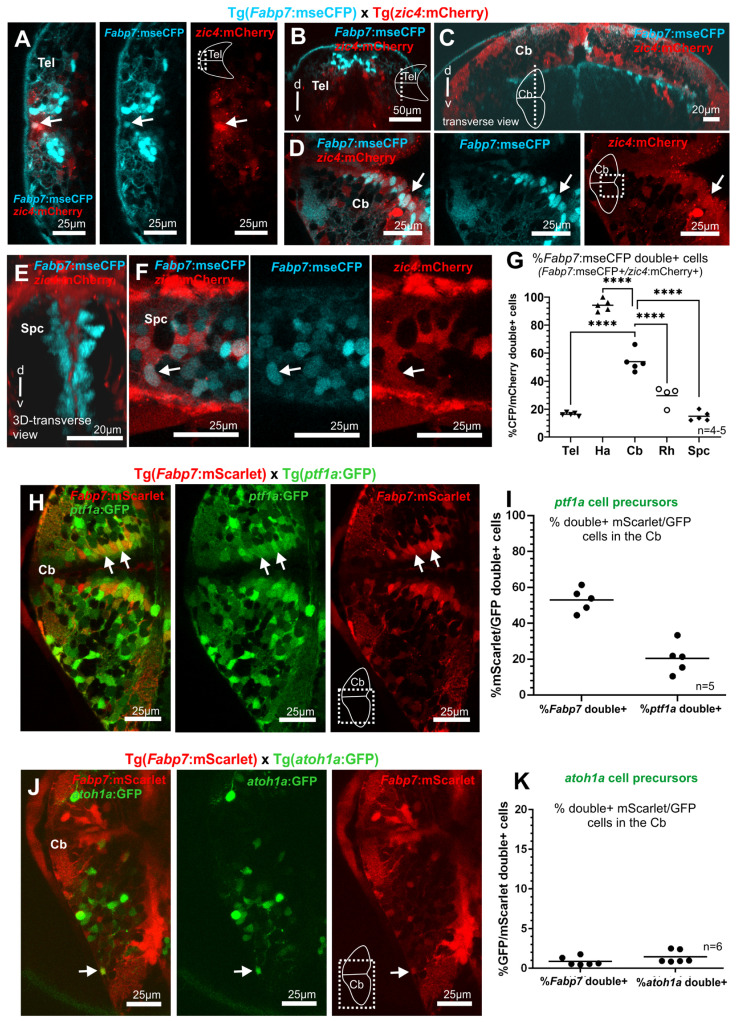
Zebrafish Bergmann glia in the cerebellum are derived from *ptf1a* enhancer-mediated fluorescent reporter expressing neural progenitor cells of the cerebellar ventricular zone. Colocalization of *Fabp7* enhancer-mediated reporter expressing cells with progenitor cells in zebrafish larvae at 5 dpf. (**A**–**G**) Pictures (**A**–**F**) and quantitative analysis of double positive cells (**G**) in double transgenic larvae Tg(*Fabp7*:mseCFP) × Tg(*zic4*:mCherry). (**H**,**I**) Pictures (**H**) and quantitative analysis of double positive cells (**I**) in double transgenic larvae Tg(*Fabp7*:mScarlet) × Tg(*ptf1a*:GFP). (**J**,**K**) Pictures (**J**) and quantitative analysis of double positive cells (**K**) in double transgenic larvae Tg(*Fabp7*:mScarlet) × Tg(*atoh1a*:GFP). Arrows point to double positive cells. Rostral is to the left. The average number and percentage of cells, corresponding to the quantification indicated in the graphs, are detailed in Supplementary Table S1. Statistical method: Ordinary one-way ANOVA, Sidák’s multiple comparison test for parametric, or normal distribution data were applied, level of significance: *p* < 0.0001 (****). Abbreviations: Cb cerebellum, Spc spinal cord, Tel telencephalon.

**Figure 7:**
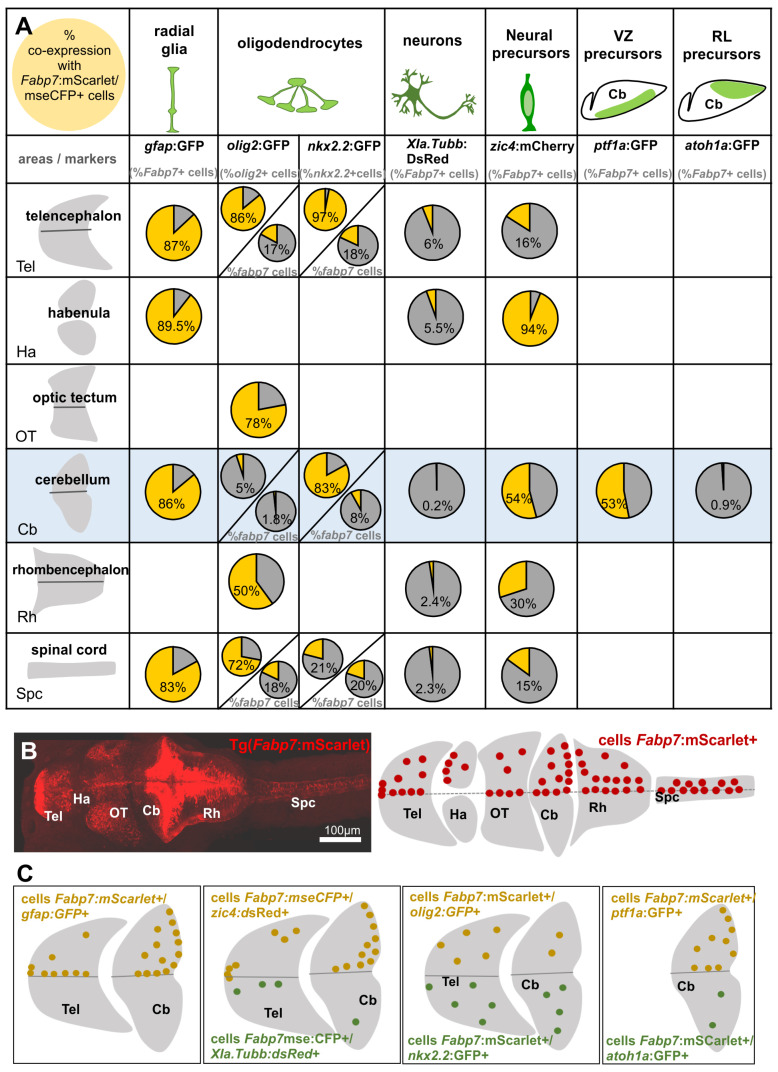
Schematic summary of the percentage and distribution of cells with fluorescent reporter protein expression mediated by the mouse *Fabp7* regulatory element with cells co-expressing other cell markers in different main areas of the brain and spinal cord. (**A**) Average of the percentage of double positive cells expressing *Fabp7* enhancer-controlled fluorescent protein expression and other glial (*gfap, olig2, nkx2.2*), neuronal (*Xla.Tubb*) cell, and cell precursor (*zic4*, *ptf1a*, *atoh1a*) markers. In the case of oligodendrocyte markers, in addition to the percentage of *Fabp7* enhancer-mediated fluorescent protein-expressing cells colabeled with *olig2* and *nkx2.2* enhancer-mediated fluorescent protein expression (lower chart), percentage of *olig2* and *nkx2.2* enhancer-mediated fluorescent protein-expressing cells colabeled with *Fabp7* enhancer-mediated fluorescent protein expression is also indicated (in order to show the ratio of oligodendrocytes derived from *Fabp7* expressing cells, upper chart). (**B**) Overview picture and schematic drawing of the distribution of *Fabp7* regulatory element expressing cells. (**C**) Schematic drawing of the distribution of cells double positive for *Fabp7* enhancer-controlled fluorescent protein expression and other specific cell markers in the telencephalon and cerebellum. Rostral is to the left. Abbreviations: *Fabp7 fatty acid binding protein* regulatory element 7, Cb cerebellum, Ha habenula, OT optic tectum, Rh rhombencephalon, RL rhombic lip, Spc spinal cord, Tel telencephalon, VZ ventricular zone.

Next, focusing on the cerebellum, the expression of *Fabp7* enhancer-regulated reporter expression in neural progenitors of the dorsal hindbrain was analyzed by using a transgenic zebrafish strain Et(*zic4*:Gal4TA4, UAS:mCherry)^hzm5^ [abbreviated name Tg(*zic4*:mCherry)]. In this transgenic strain, the red fluorescent protein mCherry is expressed under the control of a regulatory element of the *zic1/zic4* genomic locus in zebrafish [23]. These zinc finger-containing transcription factors are expressed early during central nervous system development and are involved in the regulation of cell proliferation in the dorsal neuroectoderm and formation of the hindbrain ventricle prior to the appearance of gliogenic and neurogenic lineages [41]. In double transgenic [Tg(*Fabp7*:mseCFP) x Tg(*zic4*::mCherry)] larvae, cells expressing both fluorescent reporter proteins (Figure 6A–F and Figure 7) were observed in all areas of the brain (as illustrated in the rostral most telencephalon, arrow in Figure 6A, and ventricular zone of the cerebellum, arrow in Figure 6D) as well as in the spinal cord (arrow in dorsal area, Figure 6F).

As *zic4*-expression is mostly confined to the dorsal part of the ventricular zone, dorsal CNS structures, such as the habenula and the cerebellum, showed the highest percentage of *Fabp7* regulatory element-controlled mseCFP fluorescent reporter protein-expressing cells also expressing *zic4 enhancer-mediated mCherry*, with ratios of 94% and 54% respectively (Figure 6C,D and Figure 7A,C). Since *zic4*-expression is hardly expressed in ventral, tegmental areas, compartments derived from both alar and basal plates showed a significantly lower average percentage of *Fabp7* regulatory element-mediated fluorescent protein-expressing cells co-expressing the *zic4* reporter such as 16% in the telencephalon, 30% in the rhombencephalon, and 14% in the spinal cord (Figure 6A–G and Figure 7A,C). Nevertheless, these findings reveal that *Fabp7* enhancer-mediated reporter protein expression is expressed very early during cerebellum development in neural progenitors and is maintained in glia but not in neuronal lineages.

#### 3.4.5. *Fabp7*-Regulatory Element Regulated Fluorescent Protein Expression is expressed in Cerebellar Bergman Glia, which are derived from Cerebellar Ventricular Zone

Specifically focusing on the cerebellum, two dorsally located germinal zones can be distinguished, the dorsal most positioned rhombic lip and the ventrally adjacent ventricular zone, which can be distinguished molecularly by their mutually exclusive expression of the transcription factor-encoding genes *atonal1a* (*atoh1a*) and *pancreas transcription factor 1* (*ptf1a*) respectively [42]. The *ptf1a* enhancer-mediated GFP expressing ventricular zone cells have been shown to give rise to important neuronal populations of the cerebellum, including Purkinje cells, eurydendroid cells, and inhibitory interneurons. Furthermore, Bergmann glia have been shown to originate from *gfap* and *ptf1a* enhancer-mediated fluorescent protein-labeled progenitor cells [43]. To further corroborate the origin of Bergmann glia, we crossed Tg(*Fabp7*:mScarlet) fish with Tg(*ptf1a*:GFP) carriers. Double transgenic larvae revealed a clear coexpression of the two fluorescent reporter proteins in about 20% of the *ptf1a* enhancer-mediated GFP expressing cells derived from the ventricular zone at 5 dpf (Figure 6H,I and Figure 7A,C).

This was consistent with our previous observations of Fabp7 immunohistochemistry staining in *ptf1a* enhancer-mediated fluorescent reporter protein-expressing cells, in which a fraction of about 10% of *ptf1a*:GFP green fluorescent cells in the zebrafish cerebellum was positive for Fabp7 expression [44]. Instead, only about 1% of *atoh1*-regulatory element-mediated fluorescent reporter protein-positive cells in Tg(*Fabp7*:mScarlet)/Tg(*atoh1*a:GFP) double transgenic larvae were double positive for both fluorescent reporters (meaning a total of only 1 to 6 double positive cells in the cerebellum of each larvae, being only of 0.9% of the total *Fabp7* enhancer-controlled fluorescent protein-expressing cell population as well), which may represent maintained *Fabp7* regulatory element-mediated reporter expression in very early neuroepithelial cells in which *atoh1a* and *ptf1a* enhancer-mediated fluorescent reporter protein expressing lineages have not been separated [45]. This *Fabp7* regulatory element-driven fluorescent protein-expressing subpopulation of *ptf1a* regulatory element-controlled fluorescent protein-expressing ventricular zone-derived cells represented about half (53%) of the *Fabp7* enhancer-mediated fluorescent reporter protein-expressing cells in the cerebellum (Figure 6H,I and Figure 7A,C) and may well represent progenitors of Bergmann glia as these double positive cells were mainly localized along the medial and caudal ventricular zone. Bergmann glia represent the almost only glia population of the cerebellum besides the few oligodendrocytes and are somewhat special as Bergmann glia differentiate into astrocytes of the cerebellum, but maintain characteristics of radial glia with radial fibers projecting to the pial surface in mammals as well as in zebrafish [5,46]. In the latter organism, they further contribute to the radial migration of granule cells during adult stages [5], which is considered a typical property of radial glia. Indeed, *Fabp7* regulatory element-controlled fluorescent protein-expressing cells from the Tg(*Fabp7*:mScarlet) reporter line are still detected in the adult cerebellum, which, as typical Bergmann glia in adult zebrafish, present the soma located at the boundary between Purkinje cell layer and granular cell layer, and ramified branching in the molecular layer (Supplementary Figure S3). Therefore, *Fabp7* regulatory element-mediated reporter protein expression is maintained in mature Bergmann glia and these cells marked by *Fabp7* regulatory element-mediated fluorescent protein expression can be found throughout the zebrafish cerebellar cortex. Currently, we cannot rule out that a small subpopulation of Bergmann glia is derived from rhombic lip progenitor cells, but our findings further confirm that Bergmann glia represent a subpopulation of cells being produced by the *ptf1a* enhancer-mediated fluorescent protein-expressing cells of the ventricular zone of the zebrafish cerebellum [43].

## 4. Discussion

So far, the prominent enhancer for studying radial glial cells in zebrafish was the regulatory element of the intermediate filament Glial fibrillary acidic protein-encoding gene *gfap*. Due to the heterogeneity of glial cells, the characterization of a new enhancer of the *Fatty acid binding protein Fabp7* from mice, as a reliable marker of proliferating and differentiating radial glial cells, provides a new tool for detecting a wider range of glial cells in zebrafish. The high degree of evolutionary conservation of the *Fabp7* genomic sequence allows its use in zebrafish in combination with a teleostian basal promoter. Its small size of only 742 bp (compared to 7 kb of the *gfap* enhancer), provides a valuable tool for further functional studies, for example, to create multi-cistronic constructs for monitoring consequences of transgene expression in radial glia.

The colocalization of the *Fabp7* regulatory element-mediated fluorescent reporter protein-expressing cells from the transgenic line Tg(*Fabp7*:mScarlet) with the immunolabeling of the anti-Fabp7 antibody supports the specificity of the new *Fabp7* promoter in zebrafish. Additional proof of the *Fabp7* promoter specificity for radial glial cells is evident by its coexpression with reporter fluorescent proteins mediated by the *gfap* promoter in a transgenic reporter strain (mi2001). Yet, it should be noted that a different transgenic GFP reporter line exists (zf167Tg) in which a BAC clone covering the genomic *gfap* locus of zebrafish has been used as a larger regulatory element to drive reporter protein expression, and both *gfap* reporter strains show slight differences in their reporter protein expression [47]. Nevertheless, the expression patterns of our new *Fabp7* regulatory element-controlled reporter lines in larvae appear to match with the distribution of the Fabp7 protein previously described within the adult zebrafish brain, in particular for cells in the ventricular zone of different adult brain regions [2,4,5]. Furthermore, the overlapping expression of the fluorescent proteins among the new different *Fabp7* regulatory element-controlled reporter lines observed in double transgenic larvae [Tg(*Fabp7*:mScarlet) vs. Tg(*Fabp7*:mClover) and Tg(*Fabp7*:mScarlet) vs. Tg(*Fabp7*:mseCFP)], argues that the Tg(*Fabp7*:mClover) and Tg(*Fabp7*:mseCFP) reporter lines are also specific for the Fabp7-expressing cells. The generation of these three *Fabp7* enhancer-controlled reporter lines, expressing reporter fluorescent proteins with different excitation and emission properties, greatly expands the possibilities of combinations with other cell type-specific transgenic reporter lines for research in zebrafish.

Despite the high degrees of overlapping expression of endogenous Fabp7 proteins detected by immunohistochemistry and fluorescent reporter protein expression mediated by the *Fabp7* regulatory element, the limitations of these reporter strains have to be mentioned as well. While the lack of perfect colabeling could be derived from the different half-lives of the zebrafish Fabp7 protein and the fluorescent reporter proteins, it is also possible that the *Fabp7* regulatory element is not representing the entire enhancer of the endogenous *Fabp7* homologs in zebrafish. If some enhancer or silencer elements are missing in the genomic fragment, small subpopulations of cells may ectopically express the fluorescent reporter or, conversely, may lack the expression of the reporter. Hence, the fluorescent protein expression in the *Fabp7* regulatory element containing transgenic zebrafish reporter lines may not fully reflect the entire spatio-temporal expression of the endogenous *Fabp7* homologs in zebrafish. For example, expression of fluorescent proteins in retinal Müller glia could not be found, and some weak expression in the retina may point to ectopic expression in retinal cells. Therefore, interpretations of findings of coexpression need to be cautiously made and should be confirmed independently.

To clarify the contribution of *Fabp7* regulatory element-controlled fluorescent protein-expressing cells to different cell populations in the zebrafish brain, coexpression analysis in double transgenic larvae with various transgenic reporter strains was performed (Figure 7A–C). These revealed that oligodendrocytes marked by *olig2* and *nkx2.2* enhancer-mediated reporter protein expression are derived from *Fabp7*-regulatory element-controlled fluorescent protein-positive radial glia. Indeed, colocalization of *Fabp7/olig2/nkx2.2* enhancer-mediated fluorescent reporter protein expression was observed in all brain areas and spinal cord. Of note, in previous studies, *olig2* enhancer-mediated fluorescent protein-expressing cells were negative for the radial glial cell line, in which fluorescent reporter protein expression is mediated by a *gfap* gene-derived regulatory element [3,48]. Yet, we recently showed that in the cerebellum, isolated cells with *olig2* enhancer-mediated fluorescent protein expression could be colabeled by immunohistochemistry against the Fabp7 protein [44]. This further suggests that oligodendrocyte precursors express Fabp7, and thus, *Fabp7* enhancer-mediated fluorescent reporter protein expression allows the discrimination of different radial glia-derived subpopulations of glial cells.

Since *Fabp7* expression is known to occur in proliferating neuroepithelial and radial glial cells, we also carried out a combined analysis with the neurogenic marker gene *zic4*. Indeed, a subset of *Fabp7* enhancer-mediated fluorescent protein-expressing cells coexpressed *zic4* enhancer-controlled fluorescent reporter protein expression, demonstrating that *Fabp7* regulatory element-mediated reporter expression can be found in early neuroepithelial cells. The long half-life of the fluorescent reporter protein allowed us to temporally follow *Fabp7* regulatory element-derived reporter protein expressing descendants. Such studies revealed that, unlike *gfap*-positive cells, despite some exceptions [7,12], *Fabp7* expression is found in neurogenic progenitors that give rise to *Fabp7/Xla.Tubb* enhancer-mediated fluorescent protein reporter double positive neurons, which escape the detection by Fabp7 immunohistochemistry [2]. Intriguingly, during the processes of neuroregeneration, cells derived from *gfap* regulatory element-controlled reporter protein expression are also strongly involved in the production of neurons as well [12].

In the cerebellum, two proliferation zones can be distinguished and marked by the mutually exclusive expression of neurogenic marker genes *atoh1a* and *ptf1a*. Here, half of the *Fabp7*-regulatory element-mediated fluorescent protein-expressing cells coexpressed the ventricular zone, marking fluorescent protein expression controlled by the *ptf1a* regulatory element, and hardly any coexpression of fluorescent reporter proteins mediated by the *Fabp7* and the *atoh1a* enhancers was found. Since Bergmann glia, the almost only glial cell population of the cerebellum, remain radially organized in the mouse cerebellum and also in the mature zebrafish cerebellum [5,46] and are known to express *Fabp7* endogenously, these Bergmann glial cells are likely to be derived from the *ptf1a* regulatory element-mediated fluorescent protein-expressing ventricular zone and not from the *atoh1a* regulatory element-mediated fluorescent protein-expressing upper rhombic lip. Since in zebrafish, two additional *atoh1* homologs exist with *atoh1b* and *atoh1c* [49], the origin of some radial glial cells from upper rhombic lip precursors cannot be completely ruled out. Nevertheless, the overlap in expression of *Fabp7* and *ptf1a* enhancer-controlled fluorescent reporter protein expression argues against the existence of a third cerebellar proliferation zone—different from the ventricular zone and upper rhombic lip—and that cerebellar glia are derived largely from the more ventrally positioned ventricular zone, which is supported by recent Fabp7 antibody staining found in cerebellar *ptf1a* enhancer-mediated fluorescent protein-expressing cells [44]. Moreover, these findings further support previous studies in zebrafish, where *gfap* regulatory element-mediated fluorescent protein-expressing glia were also detected to coexpress the *ptf1a* enhancer-controlled fluorescent protein expression in the cerebellar ventricular zone [43]. Indeed, in mammalians, astrocytes derive mostly from ventricular neuroepithelia as well [50]. Our findings of different glial cell types and neuronal cells derived from *Fabp7*-regulatory element-mediated fluorescent reporter protein-expressing cells further confirm the previously described heterogeneity within radial glial cells [2].

Furthermore, the newly generated *Fabp7* enhancer-controlled transgenic reporter lines provide helpful tools for developmental and regeneration studies by in vivo imaging in transparent zebrafish larvae. The stability of the fluorescent reporter proteins allows for detecting descendants of *Fabp7* enhancer-mediated fluorescent protein-expressing cells until their differentiation and could be used for comparing neuroepithelial and radial glia cell contributions under conditions of neurological diseases and processes of neuroregeneration. For example, the effect of Purkinje cell degeneration in the genetic zebrafish models of human neurodegenerative diseases Spinocerebellar Ataxia Type 1 and Type 13 could be analyzed in double transgenic specimens [51,52]. In addition, zebrafish have recently been shown to recover from specific ablation of cerebellar Purkinje cells, yet the contribution of radial glia as potential progenitors of cerebellar Purkinje cell regeneration has remained unclear [44] due to the lack of suitable genetic tools presented here. Similarly, the response of radial glia to local wounding or large injuries in the central nervous system [1,53,54,55], for example, in increasing proliferation, extending membrane protrusions, phagocytosing cellular debris, or migrating towards injury sites could be studied in vivo using bioimaging approaches. Thus, a number of informative applications could be foreseen for the radial glia reporter strains established here.

Moreover, the evolutionary conservation of the regulation of the mouse genomic *Fabp7* enhancer element in the teleost zebrafish further promises to hold true for other recently established teleostian model organisms such as *Danionella cerebrum* and *Notobranchius furzeri* [56,57]. Since these model organisms are accessible for genetic manipulations such as the generation of transgenic strains [58,59,60], genetically targeting radial glia in these models may allow us to further understand the contribution of radial glia to processes of circuitry formation, plasticity, and function in the teleost brain using adult in vivo imaging of *Danionella cerebrum* [61] or to address the contribution of radial glia to processes of central nervous system aging in *Notobranchius furzeri* [62]. Therefore, the *Fabp7* regulatory element-mediated reporter strains and the characterization of the *Fabp7* regulatory element represent powerful tools for in vivo research on glial cells in various fish model organisms.

## Data Availability

Data are contained within the article. The data presented in this study are available on request from the corresponding authors.

## Supplementary Materials

The following supporting information can be downloaded at https://www.mdpi.com/article/10.3390/cells13131138/s1, Figure S1: Sequence of mouse regulatory element from the genomic *Fabp7* locus from *Xho*I until *Sbf*I followed by the adenoviral E1b basal promoter until *Eco*RI; Figure S2: Comparison of expression fluorescent protein expression patterns in Tg(*Fabp7*:mClover), (*Fabp7*:mScarlet) (*Fabp7*:mseCFP) transgenic zebrafish larvae at 5 dpf; Figure S3: Expression of *Fabp7* regulatory element-driven mScarlet reporter protein expression in the adult brain from the Tg(*Fabp7*:mScarlet) transgenic reporter line; Table S1: Table showing average number and percentage ± standard deviation (SD), of cells expressing the fluorescent reporter proteins from the *Fabp7* transgenic reporter lines, and double positive cells from crosses of double transgenic offspring with other cell type specific transgenic reporter zebrafish lines, as well as co-labelling with the endogenous Fabp7 protein; corresponding to the quantification shown in the graphs from Figure 2, Figure 3, Figure 4, Figure 5 and Figure 6.
