# Supplementary material for: In Vivo Monitoring of Fabp7 Expression in Transgenic Zebrafish
Source: Cells. 2024 Jul 2;13(13):1138. doi: 10.3390/cells13131138 (PMC11240397; doi:10.3390/cells13131138)
Supplement: Supplementary file 1 [file cells-13-01138-s001.zip › cells-2894056-supplementary.pdf]

```
CTCGAGGTCG AGGAGGTGAA GGAAAAAAAT CTCCTCTTTG TTTCTCTAAC 50
TCATTAATGA ATTTTAAGGG CACTCTGTAA GGTTCCTTTC CCATTCTGGT 100
CTGGTTCGTA CATTCTGAGA AACACACTGT GTTTGTGTTG AGAGTTGGCT 150
CCCTAGCTAC ACTGTCTGTC ACATTGATGC TCTGAGTAGG GACAGGGTTC 200
ATCTAGGAAA TATATTTTCA CTCACACTCT GTATCTTTTC CTAGTTTGGC 250
ATATTCTAGT CTGCATTTGG CTCTCTGTTT AAATATAAAA GAAAACTAAA 300
ACACACCCTT CAGACGCCTA TGTCTGAAAA ATCTGGCATT TCCGTGGGTT 350
TTTCTTTAAG GAGGCCTTCA TTTGTAACCA ACACCATGCT CTCCTTAAGG 400
AAATCAATCT CAATGCCCTA TTATCCTTCC CTTTTCTTTC CTCCCAGTTT 450
GAGGCTGCAG TTGCCTTTTT TTTTCTTATC CCCTGCTGAA CCTGAAAAAC 500
CCTCTCTTTT CTACAGTTTT CTGTTCCCAG GCCCCGCTGA CTTCCTTTAG 550
AGCATGGGGG GGGGGGGATC AGGATTGTGA TGTGTGAACT GGGAGGATCT 600
TGACCTACTC CGCTAACCCA GTGGCCTGAG CAAATCACAA GGAGGATTGG 650
AGCCATCTGC CCAGCCCCTC CCCCACGGCA GCCTGCTGGA AAGAGACAAG 700
TTAGTCATTC AAATGATTGG CTTTTTGCCC GCAACCTGCA GGCTAGAGGG 750
TATATAATGG ATCCCATAGC TTTTCAACCT CACTTTGAGC TCCTCCACAC 800
TGAATTC
```

Figure S1. Sequence of mouse regulatory element from the genomic *Fabp7* locus from *Xho*I until *Sbf*I followed by the adenoviral E1b basal promoter until *Eco*RI. The respective restriction sites are underlined.

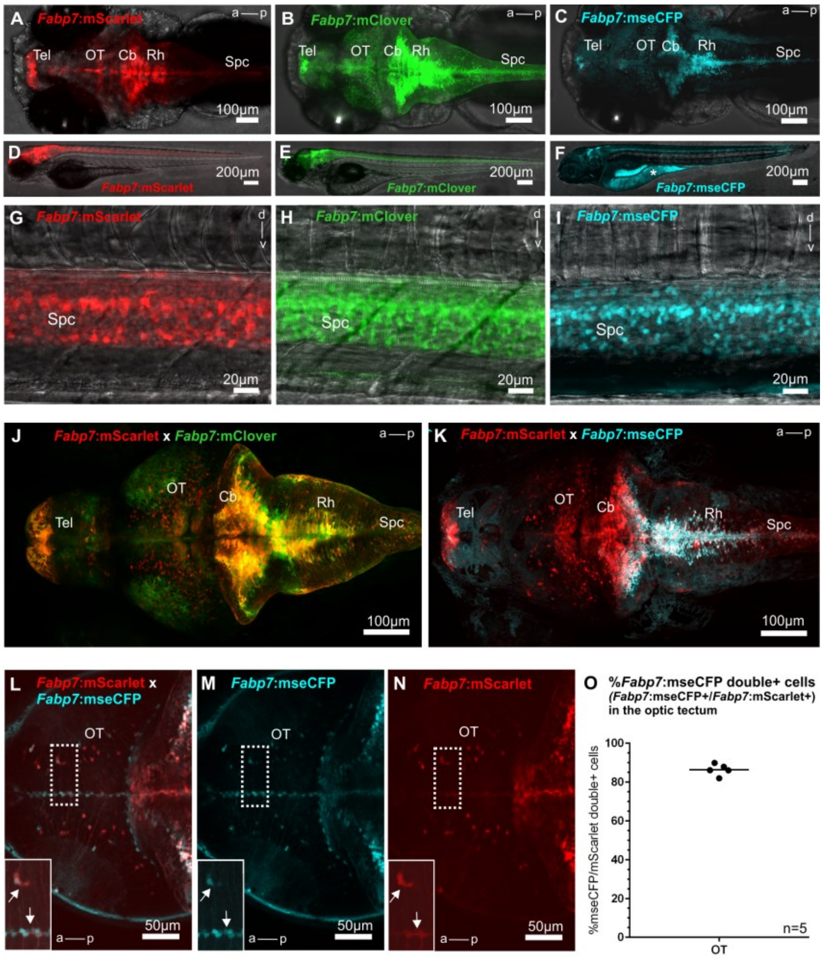

Figure S2: Single and double transgenic *Fabp7* enhancer-mediated fluorescent reporter protein expression in zebrafish larval retina at 5dpf showing the overlapping expression pattern of the three *Fabp7* reporter transgenic lines. (A-I) Overview from dorsal view of the whole brain (A-C) and lateral view of the entire larvae (D-F), and detail images of the spinal cord from lateral view (G-I), from the Tg(*Fabp7*:mScarlet), Tg(*Fabp7*:mClover), and Tg(*Fabp7*:mseCFP) transgenic larvae. (J-N)

Overview of the whole brain (J-K) and detail of the optic tectum (L-N) from dorsal view of the Tg(Fabp7:mScarlet) x Tg(Fabp7:mClover) (J) and Tg(Fabp7:mScarlet) x Tg(Fabp7:mseCFP) (K-N) double transgenic larvae showing the overlapping expression pattern of both fluorescent proteins. (O) Graph showing the degree of overlapping expression of both fluorescent proteins in double transgenic larvae in the optic tectum, indicating the percentage of mseCFP fluorescent cells also showing mScarlet fluorescence [average number ± standard deviation (SD) of mseCFP positive cells: 519,8 ± 71,07; average number ± SD of mseCFP/mScarlet double positive cells: 449,2 ± 63,84; average percentage ± SD of mseCFP/mScarlet double positive cells: 86,398 ± 2,93]. Arrows indicate double positive cells. Asterisk (*) in E indicates autofluorescence of for the mseCFP emission range in the yolk. Abbreviations: a anterior, Cb cerebellum, d dorsal, OT optic tectum, p posterior, Rh rhombencephalon, Spc spinal cord, Tel telencephalon, v ventral.

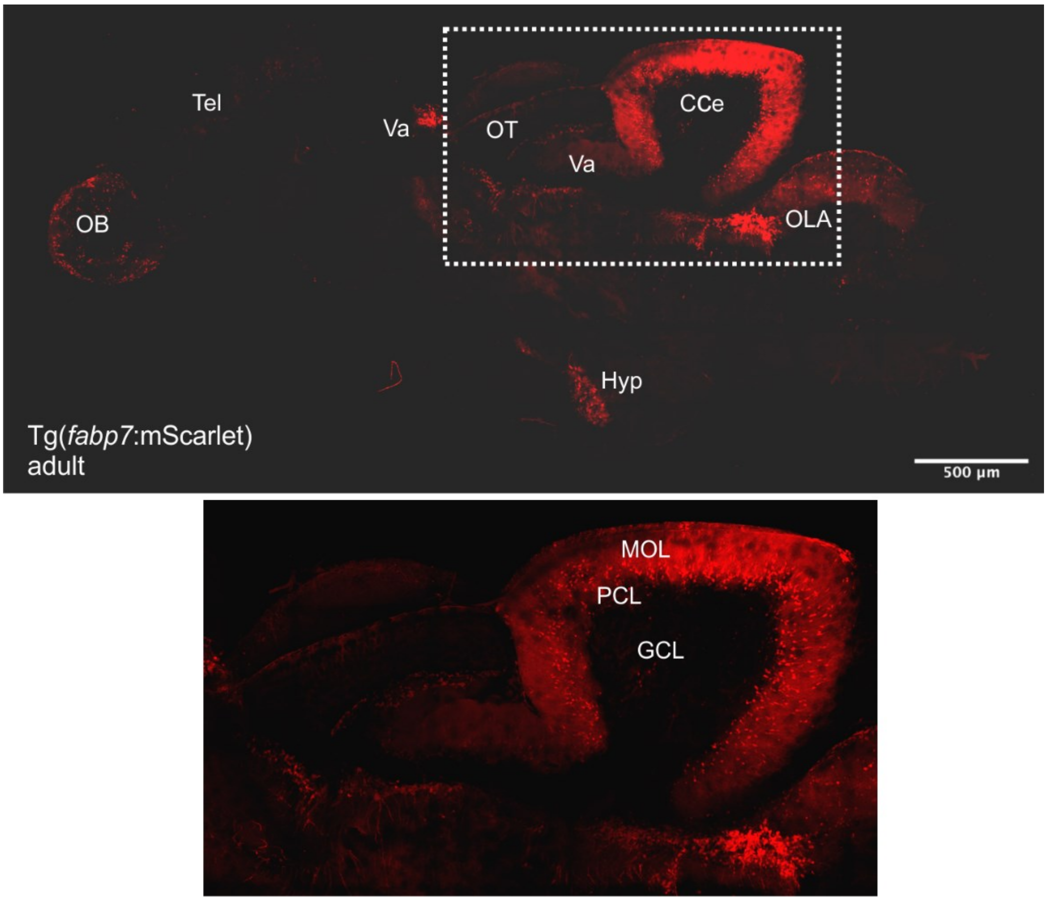

Figure S3. Expression of *Fabp7* regulatory element mediated reporter expression in the adult brain from the Tg(*Fabp7*:mScarlet) transgenic reporter line. Sagittal section showing an overview of the entire brain, and detail image of the cerebellum.

Table S1. Table showing average number and percentage ± standard deviation (SD), of cells expressing the fluorescent reporter proteins from the *Fabp7* transgenic reporter lines, and double positive cells from crosses of double transgenic offspring with other cell type specific transgenic reporter zebrafish lines, as well as co-labelling with the endogenous Fabp7 protein; corresponding to the quantification shown in the graphs from Figures 2–6.

**FIGURE-2G (n=4-5)**	**average number ± SD**	**average number ± SD**	**average percentage ± SD**
	***Fabp7*:mseCFP+ cells**	***Fabp7*:mseCFP/ mScarlet double+ cells**	**% *Fabp7*:mseCFP/ mScarlet double+ cells**
cerebellum	198,8	180,8	90,63
rhombencephalon	736,8	675,2	92,33
spinal cord	248,8	223,8	89,84

FIGURE-3E (n=5-6)	average number ± SD	average number ± SD	average percentage ± SD
	Fabp7:mScarlet+ ce-lls	*Fabp7*:mScarlet/ anti-FABP7 double+ cells	% *Fabp7*:mScarlet/ anti-FABP7 double+ cells
telencephalon	177,33±40,21	157,17±37,54	88,59±5,47
habenula	25,67±1,75	24,83±2,04	96,72±3,97
optic tectum	190,67±37,28	141,83±32,05	74,68±10,7
cerebellum	211,33±19,81	193,17±24,29	91,92±4,31
rhombencephalon	712,77±121,54	678,17±126,08	95,04±2,58
**FIGURE-3I (n=5)**	**average number ± SD**	**average number ± SD**	**average percentage ± SD**
	Fabp7:mScarlet+ ce-lls	*Fabp7*:mScarlet/ *gfap*:GFP double+ cells	% *Fabp7*:mScarlet/ *gfap*:GFP double+ cells
telencephalon	135,2±59,64	122,4±63,54	87,04±10,29
habenula	23,8±6,87	21,2±5,63	82,94±4,01
cerebellum	189,4±26,102	162,8±20,19	86,102±2,31
spinal cord	175±21,71	144,6±13,45	82,94±4,01
**FIGURE-4L (n=5)**	**average number ± SD**	**average number ± SD**	**average percentage ± SD**
	olig2:GFP+ cells	*olig2*:GFP/ *Fabp7*:mScarlet double+ cells	% *olig2*:GFP/ *Fabp7*:mScarlet double+ cells
telencephalon	26,6±5,68	23±5,83	85,78±5,27
optic tectum	101±6,96	78,6±3,36	78,2±7,45
cerebellum	97,4±29,64	4±1,87	4,71±3,79
rhombencephalon	83,6±10,31	41,4±9,63	50,41±13,93
spinal cord	66,8±26,46	45,8±19,1	72,36±19,74
**FIGURE-4M (n=5-6)**	**average number ± SD**	**average number ± SD**	**average percentage ± SD**
	nkx2.2:GFP+ cells	*nkx2.2*:GFP/ *Fabp7*:mScarlet double+ cells	% *nkx2.2*:GFP/ *Fabp7*:mScarlet double+ cells
telencephalon	25,5±4,23	24,83±3,87	97,56±2,97
cerebellum	17,42±4,24	14,43±3,1	83,27±5,12
spinal cord	256,8±76,23	55,4±15,98	21,84±3,46
**FIGURE-5D (n=5-6)**	**average number ± SD**	**average number ± SD**	**average percentage ± SD**
	Fabp7:mseCFP+ ce-lls	*Fabp7*:mseCFP/ *XIa.Tubb*:DsRed double+ cells	% *Fabp7*:mseCFP/ *XIa.Tubb*:DsRed double+ cells
telencephalon	166,67±48,03	11,8±4,82	6,41±2,68
habenula	28,17±7,63	2±3,52	5,57±9,49
cerebellum	145,33±49,64	0,33±0,82	0,24±0,59

rhombencephalon	888±93,12	21,83±10,91	2,42±1,129
spinal cord	202,4±4,88	4,6±2,07	2,27±1,01
**FIGURE-6G (n=4-5)**	**average number ± SD**	**average number ± SD**	**average percentage ± SD**
	***Fabp7*:mseCFP+ ce-lls**	***Fabp7*:mseCFP/ *zic4*:mCherry double+ cells**	**% *Fabp7*:mseCFP/ *zic4*:mCherry double+ cells**
telencephalon	145,4±19,37	23,8±3,35	16,39±1,21
habenula	21±3,54	19,8±3,56	94,21±4,129
cerebellum	282,2±35,7	151,8±24,65	53,89±7,37
rhombencephalon	478,25±34,68	142±35,74	29,7±6,7
spinal cord	318,2±98,79	46,8±17,06	14,94±3,44
**FIGURE-6I (n=5)**	**average number ± SD**	**average number ± SD**	**average percentage ± SD**
	***Fabp7*:mScarlet**	***Fabp7*:mScarlet/ *ptf1a*:GFP double+ cells**	**% *Fabp7*:mScarlet/ *ptf1a:*GFP double+ cells**
cerebellum (half)	106,8±43,23	58,4±29,53	52,93±6,58
**FIGURE-6K (n=6)**	**average number ± SD**	**average number ± SD**	**average percentage ± SD**
	***Fabp7*:mScarlet**	***Fabp7*:mScarlet/ *atoh1a*:GFP double+ cells**	**% *Fabp7*:mScarlet/ *atoh1a*:GFP double+ cells**
cerebellum	166±22,08	3,17±2,23	0,88±0,54
**FIGURE-7A (n=5)**	**average number ± SD**	**average number ± SD**	**average percentage ± SD**
	***Fabp7*:mScarlet**	***Fabp7*:mScarlet/ *nkx2.2*:GFP double+ cells**	**% *Fabp7*:mScarlet/ *nkx2.2*:GFP double+ cells**
telencephalon	134,6±14,67	24,83±3,87	18,13±2,19
cerebellum	187±38,83	14,43±3,1	7,84±2,49
Spinal cord	285,4±34,77	55,4±15,98	19,79±7,07
**FIGURE-7A (n=5)**	**average number ± SD**	**average number ± SD**	**average percentage ± SD**
	***Fabp7*:mScarlet**	***Fabp7*:mScarlet/ *olig2*:GFP double+ cells**	**% *Fabp7*:mScarlet/ *olig2*:GFP double+ cells**
telencephalon	139,6±18,72	23±5,83	16,76±5,27
cerebellum	235,2±67,69	4±1,87	1,82±1,11
Spinal cord	257±71,52	45,8±19,1	18,11±7,57
